# The Prognostic Role of Programmed Ventricular Stimulation in the Risk Stratification of Sudden Cardiac Death

**DOI:** 10.31083/j.rcm2405152

**Published:** 2023-05-19

**Authors:** Michele Iavarone, Anna Rago, Riccardo Molinari, Antonello D’Andrea, Martina Nesti, Saverio Muscoli, Giuseppe Mascia, Vincenzo Russo

**Affiliations:** ^1^Cardiology Unit, Department of Translational Medical Sciences, University of Campania “Luigi Vanvitelli”, AORN Ospedali dei Colli Monaldi Hospital, 80131 Naples, Italy; ^2^Unit of Cardiology and Intensive Coronary Care, Umberto I Hospital, 84014 Nocera Inferiore, Italy; ^3^Cardiology Unit, Fondazione Toscana Gabriele Monasterio, 56124 Pisa, Italy; ^4^Division of Cardiology, Fondazione Policlinico Tor Vergata, 00133 Rome, Italy; ^5^Cardiovascular Disease Unit, Department of Cardiology, IRCCS Ospedale Policlinico San Martino, 16132 Genova, Italy

**Keywords:** programmed ventricular stimulation, sudden cardiac death, risk stratification

## Abstract

Sudden cardiac death (SCD) is one of the leading causes of cardiovascular death in general population. SCD primary prevention requires the correct selection of patients at increased risk who may benefit from implantable cardioverter-defibrillator (ICD). Despite several non-invasive arrhythmic risk indexes are available, their ability to stratify the SCD risk among asymptomatic patients with cardiac disease at increased arrhythmic risk is debated. The programmed ventricular stimulation (PVS) is an invasive approach historically used for SCD risk stratification in patients with acquired or inherited cardiac disease and is currently included in international guidelines. Aim of this review is to summarize all available data about the role of PVS for the SCD risk stratification in different clinical settings.

## 1. Introduction

The programmed electrical stimulation and the intracardiac activation mapping were introduced in 1967 for studying the re-entry arrhythmias in Wolff-Parkinson-White Syndrome [1]; and in 1972 for the evaluation of ventricular arrhythmias (VAs) [2]. The programmed ventricular stimulation (PVS) was initially performed to guide pharmacological therapy in patients with recurrent sustained ventricular arrhythmias (VAs) [3] or cardiac arrest (CA) [4]; in this clinical setting, the PVS showed an increased prognostic value compared to the non-invasive approach [5, 6]. Over the years, several studies investigated the role of PVS in the risk stratification of sudden cardiac death (SCD) in patients with recent myocardial infarction (MI) [7, 8, 9, 10] or with history of VAs, including non-sustained forms [11, 12]. In 1999 the MUSTT trial [13] demonstrated that role of PVS in identifying high-risk patients with coronary artery disease (CAD) who benefit from antiarrhythmic therapy, including implantable cardiac defibrillator (ICD) [14]. Actually, several stimulation protocols, different definitions of positive response at PVS and heterogeneous study populations led to doubts about the prognostic role of PVS [15, 16, 17]. The aim of the present review is to summarize all available data about the role of PVS for the SCD risk stratification in different clinical settings.

## 2. Coronary Artery Disease

Coronary heart disease is the most common cardiac condition associated with SCD [18, 19]. Patients with CAD are considered in need of ICD implantation for high SCD risk when left ventricular ejection fraction (LVEF) is ≤30% or ≤35%, New York Heart Association (NYHA) class is I and II–III respectively, despite at least 3 months of optimal medical therapy (OMT). CAD patients with LVEF ≤40% despite at least 3 months of OMT and non-sustained ventricular tachycardia (NSVT) should be stratified with PVS [20, 21]. This indication is based on the results of the randomized controlled MUSTT trial [14] that evaluated the PVS role in 2202 CAD patients with LVEF ≤40% and NSVT. Patients with inducible VAs were randomly assigned to receive PVS-guided antiarrhythmic therapy (first-line drugs, second-line drugs or ICD implant) or no therapy. The study demonstrated that PVS-guided antiarrhythmic drug treatment had a lower incidence of the primary endpoint, a composite of cardiac arrest and arrhythmic death, compared to no-treatment arm (12% vs 25%, after 24 months; and 18% vs 32%, after 60 months, *p* = 0.043, HR 0.73) [22].

The subgroup analysis of patients treated with antiarrhythmic drugs vs ICD showed that the entire benefit of PVS-guided therapy arm was only due to ICD therapy [22]. However, it should be noted that, even if the MUSTT trial enrolled patients with LVEF ≤40%, the average LVEF of the study population was 30%. Moreover, the improvement in the revascularization techniques and in pharmacological therapies reduced the incidence of SCD in heart failure patients [23] and the rates of appropriate shocks over time [24]. The role of ventricular fibrillation (VF) inducibility as a predictor of SCD in CAD patients is still debated [25]. According to American Guidelines [20] and MUSTT study [14] the PVS was considered positive when VF is induced; in contrast, the current European guidelines [21] consider only sustained monomorphic ventricular tachycardia (SMVT) as PVS positive result.

Primary prevention trials did not include patients with CAD and LVEF >40%, because they were commonly considered at lower risk of VAs. However, in the current era of early revascularization and OMT, most SCD or CA events occur in patients with preserved or mildly reduced ejection fraction [26], yielding an annual incidence of 0.6% [27].

The PRESERVE-EF, a multicenter prospective observational cohort study, investigated the role of a two-step approach for risk stratification of 575 post-MI patients (66.3% ST-elevation myocardial infarction (STEMI) and 33.7% non-ST-elevation myocardial infarction (NSTEMI)) with LVEF ≥40% [28]. The first step was evaluating the presence of at least one non-invasive risk factor among frequent premature ventricular complexes (PVCs), NSVT, late potentials, prolonged corrected QT interval, increased T-wave alternans, reduced heart rate variability, abnormal deceleration capacity with abnormal turbulence. In presence of at least one risk factor, patients underwent PVS and, if positive for sustained monomorphic ventricular tachycardia (SMVT), an ICD was implanted. During a mean follow-up of 32 months, 9 out of 41 inducible patients experienced an appropriate ICD therapy (shock in 7 cases and ATP in 2 cases); moreover, none patients with negative PVS met the primary endpoint. The PRESERVE-EF study suggested that the two-step approach is useful to detect post-MI patients with LVEF ≥40% at high risk of major arrhythmic events that can be effectively addressed with and ICD. However, it is not still clear if appropriate ICD therapies can be considered a reliable surrogate of SCD; therefore, there were no specific recommendations for SCD prevention in this subgroup of patients [20, 21].

Before the fortieth day after MI, the ICD implantation in SCD primary prevention is contraindicated, since two randomized trials showed no benefit on overall mortality when ICD was implanted early after MI [29, 30].

The ongoing PROTECT-ICD randomized trial [31] is currently evaluating whether PVS may identify a subgroup of patients with LVEF ≤40% that benefit from ICD therapy in the early phase after MI (NSTEMI or STEMI). Patients within 2 and 40 days after MI with LVEF ≤40% are randomized 1:1 to conventional arm or invasive arm including PVS and ICD implantation in patients with inducible SMVT. Moreover, this study will evaluate if cardiac magnetic resonance imaging (CMR) may have additional risk stratification capability in this population. Table 1 (Ref. [14, 28, 31]) summarizes the main studies about the prognostic role of PVS in CAD patients.

**Table 1.** **Programmed ventricular stimulation in patients with coronary artery disease**.

Authors	Year	Study protocol	Patients (n)	Stimulation protocol	Inducibility	Conclusions
Buxton *et al*. [14]	1999	Clinical trial	2202	Up to three extrastimuli from RVA and RVOT	SMVT by any method of stimulation or PVT/VFL/VF by one or two extrastimuli	PVS-guided treatment reduces SCD risk (HR 0.73)
Gatzoulis *et al*. [28]	2019	Prospective observational study	575	Up to three extrastimuli from RVA and RVOT	SMVT/PVT/VFL	22% PPV
						100% NPV for major arrhythmic events
Zaman *et al*. [31]	2016	Clinical trial	Enrolling	Up to four extrastimuli from RVA	SMVT	Ongoing

NPV, negative predictive value; PPV, predictive positive value; PVS, programmed ventricular stimulation; PVT, polymorphic ventricular tachycardia; RVA, right ventricular apex; RVOT, right ventricular outflow tract; SCD, sudden cardiac death; SMVT, sustained monomorphic ventricular tachycardia; VF, ventricular fibrillation; VFL, ventricular flutter.

In conclusion, the PVS has a clear role in the risk stratification of CAD patients with LVEF ≤40% and history of NSVT; moreover, it may be considered to stratify CAD patients with LVEF >40% and at least one additive risk factor among the following: frequent PVCs, NSVT, late potentials, prolonged QTc, increased T-wave alternans, reduced heart rate variability, abnormal deceleration capacity with abnormal turbulence. If PVS may identify a subgroup of patients with LVEF ≤40% that benefit from ICD therapy in the early phase after MI (NSTEMI or STEMI) is currently under investigation.

## 3. Non-Ischemic Cardiomyopathy

Patients with non-ischemic cardiomyopathy (NICM), NYHA class II–III and LVEF ≤35%, despite at least 3 month of OMT, are considered at increased SCD risk [20, 21, 32, 33] and ICD implantation is recommended by the current guidelines [20, 21].

The DANISH trial has randomized 1116 NICM patients with left ventricular ejection fraction ≤35% to receive ICD or usual clinical care in order to evaluate the overall survival benefit of prophylactic ICD implantation. An age-dependent association between ICD and mortality was shown with a survival benefit for patients <70 years, that was not confirmed in those ≥70 years.

The SCD risk stratification of NICM patients with LVEF between 35% and 50% is still a challenging clinical issue and PVS is supported only by expert consensus. In patients with syncope, the PVS should be considered when the loss of consciences remains unexplained or presumed arrhythmic after non-invasive assessment (Class IIa, level of evidence C) [20, 21]. Moreover, the PVS-inducibility of SMVT is considered a risk marker of VAs and ICD implant is recommended in NICM with LVEF <50% and at least another risk factor among the following: history of syncope, late gadolinium enhancement on cardiac magnetic resonance (CMR), pathogenic mutations in high-risk genes (*LMNA*, *PLN*, *FLNC*, or *RBM20*) [21].

The predictive role of PVS in SCD stratification of NICM patients has been first shown by Gatzoulis *et al*. [34]; in a cohort of 158 patients followed for 46.9 months, the first time ICD activation rate was significantly higher in inducible compared to non-inducible patients (73.2% vs 17.9%; log-rank *p* = 0.001) with no significative difference in SCD and overall mortality.

A recent meta-analysis, including 45 studies and 6088 NICM patients, with the purpose to estimate the performance of 12 commonly reported risk stratification tests as predictors of arrhythmic events, suggested that PVS was the most specific (87.1%) but less sensible (28.8%) test for the SCD risk stratification [35].

The ongoing multicenter, prospective observational ReCONSIDER study [36] is evaluating the potential of a multifactorial approach, in which non-invasive risk factors are combined with PVS to achieve arrhythmic risk stratification of NICM patient with LVEF ≤50%. Patients are divided in 2 groups: patients with LVEF between 35% and 50% in group A and patients with LVEF ≤35% in group B. A further subdivision in 6 subgroups is performed according to a two-step approach. The first step includes the identification of non-invasive risk factors including suspected high-risk syncope and/or presyncope, dilated left ventricle, late gadolinium enhancement on cardiac MRI, frequent PVCs, NSVT, late potentials, prolonged QTc interval, increased T-wave alternans, reduced heart rate variability, abnormal deceleration capacity with abnormal turbulence. The second step is represented by induction of any VA at PVS, following the protocol described by Gatzoulis *et al*. in 2013 [34]. All patients in group B and patients in subgroup A3 (patients in group A with at least one risk factor and a positive response at PVS) will receive an ICD or a cardiac resynchronization therapy defibrillator. Primary endpoint is the occurrence of major arrhythmic events including sustained VT/VF, ICD activation and SCD. Table 2 (Ref. [34, 36]) summarizes the main studies about the prognostic role of PVS in NICM patients.

**Table 2.** **Programmed ventricular stimulation in patients with non-ischemic cardiomyopathy**.

Authors	Year	Study protocol	Patients (n)	Stimulation protocol	Inducibility	Conclusions
Gatzoulis *et al*. [34]	2013	Prospective observational study	158	Up to three extrastimuli from RVA and RVOT	Sustained VT or VF	Increased risk of ICD activation
Gatzoulis *et al*. [36]	2021	Prospective observational study	Enrolling	Up to three extrastimuli from RVA and RVOT	Sustained VT or VF	Ongoing

ICD, implantable cardiac defibrillator; RVA, right ventricular apex; RVOT, right ventricular outflow tract; VT, ventricular tachycardia; VF, ventricular fibrillation.

In conclusion, PVS may be useful to predict the risk of VAs in NICM patients and is currently recommended in patients with unexplained syncope or with at least one non-invasive risk factor evidenced by genetic testing or CRM.

## 4. Hypertrophic Cardiomyopathy

Except in the setting of unexplained syncope after non-invasive evaluation, the predictive role of PVS in patients with hypertrophic cardiomyopathy (HCM) is still unclear [37], and no guidelines consider it for the SCD risk stratification in this population [38, 39].

However, the role PVS was investigated in a recent prospective observational study by Gatzoulis *et al*. [40] recruiting 203 consecutive HCM patients with at least one non-invasive risk factor for VAs including family history SCD in a first degree relative, a recent episode of unexplained syncope and/or presyncope, NSVT, hypotensive or attenuated blood pressure response to exercise, maximal wall thickness ≥30 mm. The study showed that the incidence of SCD or appropriate ICD therapies were significantly higher (24% vs 0.8%, *p *< 0.001) in the PVS inducible patients compared to those non-inducible; the PVS sensitivity and specificity was 95% and 67.2%, respectively with a positive predictive value (PPV) = 24% and negative predictive value (NPV) = 99.2% [40]. These results appear to contradict the earlier findings concerning the role of PVS in HCM SCD risk stratification; however, some of historical studies included relatively small cohorts of patients and did not correlate the PVS positivity with patients’ clinical outcomes. Since in the study by Gatzoulis *et al*. [40] the CMR was not performed, further studies are necessary to evaluate PVS may be integrated in modern algorithms of SCD risk stratification including CMR and genetic testing.

## 5. Arrhythmogenic Cardiomyopathy

The role of PVS in SCD risk stratification of patients with arrhythmogenic cardiomyopathy (ACM) is still debated. American guidelines on VAs and SCD prevention did not consider PVS in the risk stratification of ACM patients [20] since a multicenter prospective observational study by Corrado *et al*. [41], including 106 ACM patients followed for 58 ± 31 months, showed the low PPV and NPV for any appropriate ICD therapy and for ICD shock, about 35% and 20%; and 70% and 74%, respectively. More recently, European guidelines recommend PVS in Class IIb for risk stratification of ACM patients with symptoms suggestive of VAs (presyncope or palpitations) [21]. Moreover, ICD implantation is recommended in symptomatic patients with moderate right and/or left ventricular dysfunction and inducible SMVT at PVS (Class IIa).

The indication of the European guidelines is based on the results of two observational studies that showed a significant predictive role of PVS in SCD risk stratification of ACM patients.

In a cohort of 84 ACM patients followed for 4.7 ± 3.4 years, Bhonsale *et al*. [42] showed that the VAs inducibility is a significant predictor of appropriate ICD interventions (HR: 4.5; 95% CI: 1.37 to 14.96; *p* = 0.013) with a PPV of 65% and a NPV of 75%. Saguner *et al*. [43] confirmed the usefulness of the inducible SMVT as predictor of appropriate ICD interventions (HR 2.52, 95% CI: 1.03 to 6.16, *p* = 0.043) in a long-term outcome (median 9.8 years) with a PPV of 65% and NPV of 72%. The high number of ACM patients with symptoms suggestive of VAs or with history of sustained VAs included in these studies may have contributed to the exclusion of PVS from American guidelines.

Recently, a multicenter retrospective observational study by Gasperetti *et al*. [44] evaluated the predictive role of PVS in 288 ACM patients with low prevalence of symptoms suggestive of VAs during a median follow-up of 5.31 years. The PVS inducibility of SMVT had a 76% sensitivity and 68% specificity in the overall cohort; with a PPV of 38.5% and a NPV of 92.6% in low/intermediate risk patients. The authors concluded that a 2-step approach integrating PVS into the risk calculator’s prediction significantly improved the prediction of arrhythmic outcomes 5 years after diagnosis beyond the ACM risk calculator. Table 3 (Ref. [41, 42, 43, 44]) summarizes main studies on PVS in patients with arrhythmogenic cardiomyopathy.

**Table 3.** **Programmed ventricular stimulation in patients with arrhythmogenic cardiomyopathy**.

Authors	Year	Study protocol	Patients (n)	Stimulation protocol	Inducibility	Conclusions
Corrado *et al*. [41]	2010	Prospective observational study	106	Up to three extrastimuli from RVA and RVOT	Sustained VT or VF	35% PPV for appropriate ICD therapy
Bhonsale *et al*. [42]	2011	Prospective observational study	84	Local protocols	Sustained VT or VF	65% PPV for appropriate ICD interventions (HR: 4.5)
Saguner *et al*. [43]	2013	Retrospective observational study	62	Up to three extrastimuli from RVA and RVOT	SMVT	65% PPV for appropriate ICD interventions (HR: 2.52)
Gasperetti *et al*. [44]	2022	Retrospective observational study	288	Up to three extrastimuli (88%) from RVA and RVOT (89%)	SMVT	38.5% PPV 92.6% NPV for 5-year sustained VAs

ICD, implantable cardiac defibrillator; NPV, negative predictive value; PPV, predictive positive value; RVA, right ventricular apex; RVOT, right ventricular outflow tract; SMVT, sustained monomorphic ventricular tachycardia; VA, ventricular arrhythmia; VT, ventricular tachycardia; VF, ventricular fibrillation.

In conclusion, the inducibility of SMVT at PVS may be considered an arrhythmic risk marker in ACM patients symptomatic for presyncope or palpitations; moreover, it may refine risk estimates, improving the decision-making process about ICD implantation in selected ACM patients. If PVS may be used in SCD risk stratification of asymptomatic ACM patients is still unclear.

## 6. Myotonic Dystrophy

The role of PVS in the risk assessment of type 1 myotonic dystrophy (MD1) is still controversial [45, 46, 47, 48]. European guidelines recommend ICD implantation in MD1 patients with palpitations highly suspicious for VA and induction of VT other than bundle branch re-entry VT (Class IIa, level of evidence C) [21]. Electrophysiological testing should be considered in MD1 patients who are older than 40 years and have supraventricular arrhythmias or extensive late gadolinium enhancement on CMR (Class IIa, level of evidence C). Moreover, the heart rhythm society consensus statement on evaluation and management of arrhythmic risk in neuromuscular disorders recommend PVS in MD1 patients with symptoms suggestive of VAs not explained by non-invasive testing (Class IIb, level of evidence B) [49]. In the ACADEMY 1, a recent prospective study including 72 MD1 patients in need of permanent pacing and underwent ICD implantation according to the results of PVS, Russo *et al*. [50] showed a low PPV (about 16%) in predicting arrhythmic events during a mean follow-up period of 44.7 ± 10.2 months; conversely, the NPV was 90%. The PVS was conducted up to three extrastimuli from both RVA and right ventricular outflow tract (RVOT); and as PVS positivity was considered the inducibility of sustained VT or VF. Considering the high incidence life-tethering arrhythmic events in DM1 patients, the decision to implant ICD should not be based exclusively on the PVS findings.

## 7. Adult Congenital Heart Disease 

Since no randomized clinical trial for SCD prevention has included patients with congenital heart disease (ACHD), the international guidelines recommendations on SCD risk stratification were extrapolated from studies on repaired tetralogy of Fallot (TOF).

According to the American guidelines, the PVS should be considered in repaired TOF patients with high-risk features and frequent VAs (frequent PVCs or NSVT) (Class IIa) [20]; in contrast, the European guidelines suggest PVS in repaired TOF patients with arrhythmia symptoms and NSVT (Class IIa) or with a combination of risk factors (Class IIb) [21]. Non-invasive risk factors which identify repaired TOF patients at high-risk of VAs are reported in Table 4.

**Table 4.** **Non-invasive risk factors for SCD in repaired TOF patients**.

Source	Year	Non-invasive risk factors
AHA/ACC/HRS Guidelines for management of patients with VAs and the prevention of SCD	2017	Prior palliative systemic to pulmonary shunts
		Unexplained syncope
		Frequent PVCs
		Atrial tachycardia
		QRS duration ≥180 ms
		Left ventricular systolic or diastolic dysfunction
		Dilated right ventricle
		Severe pulmonary regurgitation or stenosis
		Elevated levels of BNP
ESC Guidelines for the management of patients with VAs and the prevention of SCD	2022	Moderate right or left ventricular dysfunction
		Extensive right ventricular scarring on CMR
		QRS duration ≥180 ms
		Severe QRS fragmentation

BNP, brain natriuretic peptide; CMR, cardiac magnetic resonance; PVCs, premature ventricular contractions; SCD, sudden cardiac death; TOF, Tetralogy of Fallot; VAs, ventricular arrhythmias.

These indications are mainly based on a multicenter retrospective observational study by Khairy *et al*. [51] which included 252 repaired TOF patients followed for 6.5 ± 4.5 years after PVS. In their study cohort, the inducibility of VT/VF at PVS showed a high sensitivity (77.4 ± 5.3%) and specificity (79.5 ± 2.9%) in predicting VAs, regardless of the patients’ symptomatology. The PVS showed a PPV and NPV of 55.2 ± 5.3% and 91.5 ± 2.2%, respectively [51]. A protocol including three extrastimuli from both RVA and RVOT and considering as positivity the inducibility of sustained VT or VF was used.

In conclusion, ACHD patients with a combination of at least 2 non-invasive risk factors (Table 4) could benefit from PVS, especially if symptomatic for VAs or with documented NSVT.

## 8. Brugada Syndrome

The role of PVS in the SCD risk assessment of patients with Brugada Syndrome (BrS) is still debated. Early observational studies suggested the high sensitivity of PVS in identifying patients at SCD increased risk, especially in asymptomatic subjects with spontaneous type 1 electrocardiographic (ECG) pattern and in those with syncope and induced- type 1 ECG pattern [52, 53].

In contrast, data from two large European registries, FINGER [54] and PRELUDE [55] including 1029 and 308 patients respectively, showed a poor capacity of PVS to predict VAs in BrS patients [54, 55, 56].

A systematic review by Sroubek *et al*. [57] including 1312 BrS patients, defined as symptomatic for syncope (33%) or asymptomatic (67%); and as spontaneous (53%) or pharmacologically induced (47%) type 1 ECG pattern, supported the role of PVS, with single or double extrastimuli, in predicting arrhythmic risk among asymptomatic spontaneous type 1 BrS patients.

Based on these results, both American and European guidelines recommended PVS up to two extrastimuli in asymptomatic patients with spontaneous type 1 ECG in class IIb and suggest ICD implantation in individuals with inducible VF in the same class of recommendation [20, 21].

Guidelines do not include recommendations for BrS patients with pharmacologically induced type 1 ECG pattern. Although this group demonstrated a relatively low SCD risk, it should not be considered insignificant [58, 59]. In the multicenter observational retrospective IBRYD study including 226 BrS patients with drug induced type 1 ECG, 4.9% of them experienced an appropriate ICD therapy or SCD during a median follow-up of 106 months [59, 60]. In a recent meta-analysis including 4.099 BrS patients followed for 4.5 years, the pooled annual incidence of major arrhythmic events was 0.65% in symptomatic and 0.21% in asymptomatic BrS patients with drug-induced type 1 ECG. The incidence of major arrhythmic events was similar in symptomatic induced type 1 ECG and in asymptomatic spontaneous type 1 ECG. Moreover, despite a low PPV (8.9% in asymptomatic; 9.6% in symptomatic), PVS demonstrated a high NPV (95% in asymptomatic; 100% in symptomatic) for SCD risk stratification in high-risk patients with drug-induced type 1 ECG [61]. Therefore, based on current evidence, performing PVS for SCD risk stratification of BrS patients with drug-induced type 1 ECG remains controversial [62] and should be guided by non-invasive risk factors [63, 64] such as unexplained syncope, genetic testing and family history of sudden cardiac death. Table 5 (Ref. [52, 53, 54, 55, 56, 59]) summarizes the main studies on PVS in BrS patients with both spontaneous and drug-induced type 1 ECG pattern.

**Table 5.** **Programmed ventricular stimulation in patients with Brugada syndrome**.

Authors	Year	Study protocol	Patients (n)	Stimulation protocol	Inducibility	Conclusions
Brugada *et al*. [52]	2003	Prospective observational study	547	Up to three extrastimuli from RVA	Sustained VT or VF	Predictive of VF or SCD
Giustetto *et al*. [53]	2009	Prospective observational study	166	Up to two extrastimuli from RVA and RVOT	Sustained VT or VF	Predictive of arrhythmic events (sustained VT, VF or SCD)
Probst *et al*. [54]	2010	Subanalysis of FINGER registry	1029	Up to three extrastimuli from RVA and RVOT	Sustained VT or VF	Not predictive of arrhythmic events
Delise *et al*. [56]	2011	Prospective observational study	320	Up to two extrastimuli from RVA and RVOT	Sustained VT or VF	Not predictive of arrhythmic events
Priori *et al*. [55]	2012	Subanalysis of PRELUDE registry	308	Up to three extrastimuli from RVA and RVOT	Sustained VT or VF	Not predictive of arrhythmic events
Russo *et al*. [59]	2021	Retrospective observational	226	Up to three extrastimuli from RVA and RVOT	Sustained VT or VF	Low PPV and a high NPV

NPV, negative predictive value; PPV, positive predictive value; RVA, right ventricular apex; RVOT, right ventricular outflow tract; SCD, sudden cardiac death; VT, ventricular tachycardia; VF, ventricular fibrillation.

## 9. Primary Electrical Diseases

PVS is not currently recommended in primary electrical disease [20, 21] since only two studies (Table 6, Ref. [65, 66]) have evaluated its role in the SCD risk stratification and both showed a poor predictive value of PVS in patients with long QT syndrome and early repolarization syndrome [65, 66].

**Table 6.** **Programmed ventricular stimulation in patients with primary electrical diseases**.

Authors	Year	Study protocol	Patients (n)	Stimulation protocol	Inducibility	Conclusions
Bhandari *et al*. [65]	1985	Prospective observational study	15	Up to three extrastimuli from RVA and RVOT	Sustained VT or VF	No prediction of arrhythmic events
Mahida *et al*. [66]	2015	Retrospective observational study	81	Up to three extrastimuli from RVA and RVOT	VF	No prediction of arrhythmic events

RVA, right ventricular apex; RVOT, right ventricular outflow tract; SVT, sustained ventricular tachycardia; VF, ventricular fibrillation.

## 10. Syncope

Programmed ventricular stimulation may be considered in patients with syncope preceded by palpitations and is recommended in patients with previous MI, regardless of LVEF, or other scar-related conditions (e.g., previous myocarditis or cardiac surgery) [67, 68].

## 11. Conclusions

The SCD risk stratification in acquired and inherited cardiac diseases remains a challenging clinical issue and the role of PVS is still debated as well as the stimulation protocol. In most studies VF is accepted as a positive result, however except for BrS, VF is not predictive of ventricular arrhythmias.

**Fig. 1.**
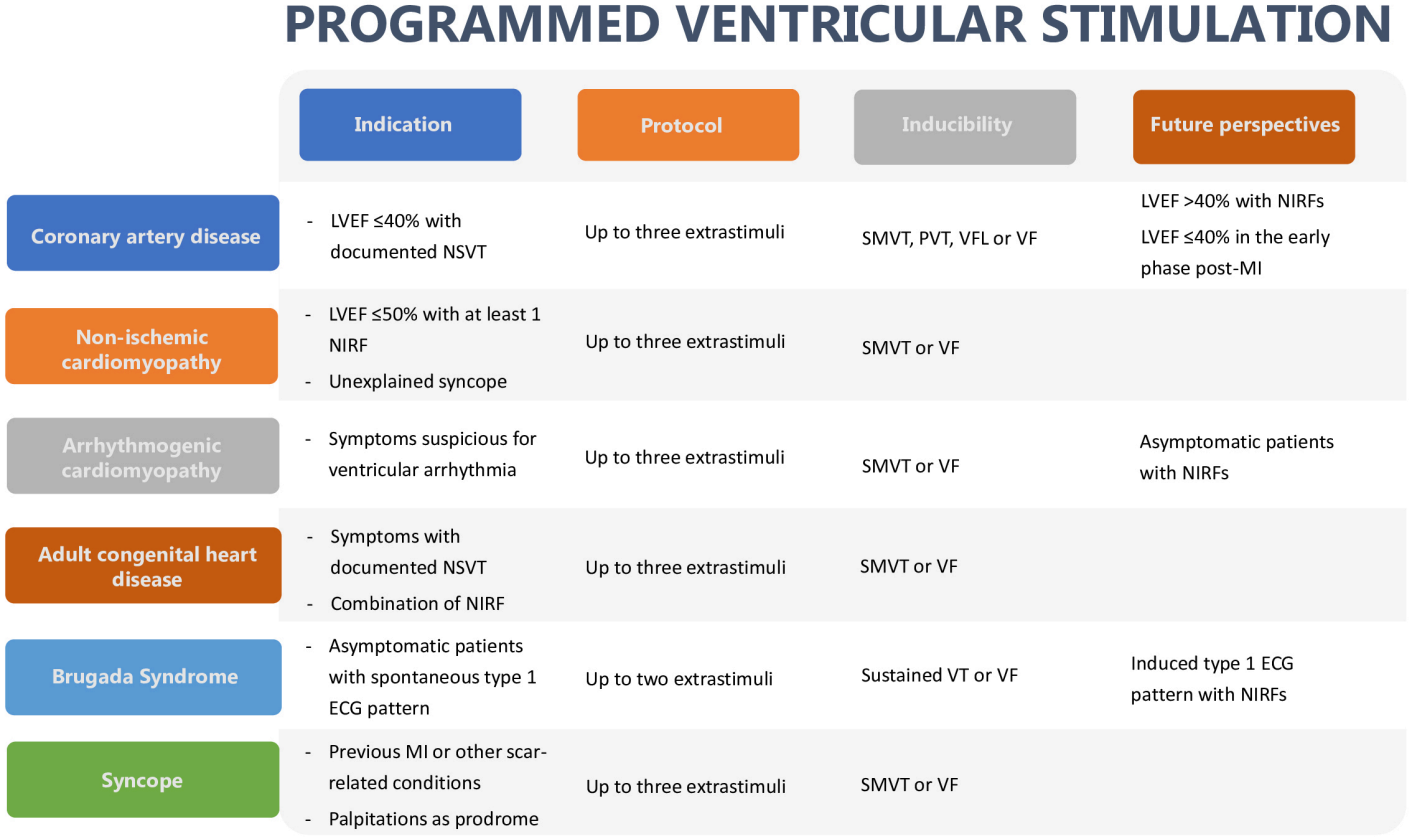
**Programmed ventricular stimulation in main clinical settings**. LVEF, left ventricular ejection fraction; MI, myocardial infarction; NIRF, non-invasive risk factor; NSVT, non-sustained ventricular tachycardia; PVT, polymorphic ventricular tachycardia; SMVT, sustained monomorphic ventricular tachycardia; VF, ventricular fibrillation; VFL, ventricular flutter.

The analysis of the available data suggests PVS is a useful tool in several clinical conditions (Fig. 1) when the non-invasive stratification identifies an intermediate risk profile; in this subset patients, the high predictive negative value supports the conservative management.

## Data Availability

The data used to support the finding of this study are available within the article.
